# Does septum resection improve reproductive outcomes for women with a septate uterus? A systematic review and meta-analysis

**DOI:** 10.3389/fendo.2024.1361358

**Published:** 2024-07-22

**Authors:** Chang Liu, Zhiqi Liao, Xueqi Gong, Yinwei Chen

**Affiliations:** ^1^ Reproductive Medicine Center, Nanjing Drum Tower Hospital, The Affiliated Hospital of Nanjing University Medical School, Nanjing, China; ^2^ Reproductive Medicine Center, Tongji Hospital, Tongji Medical College, Huazhong University of Science and Technology, Wuhan, China

**Keywords:** hysteroscopy, embryo transfer, pregnancy, uterine cavity, assisted reproduction technique

## Abstract

**Objective:**

To investigate whether incising the septum facilitates reproductive outcomes for patients with a septate uterus compared to expectant management.

**Methods:**

Research was retrieved from three electronic databases: PubMed, Embase, and the Cochrane Library, with no time or language restrictions. Two authors independently selected the articles and extracted data regarding study characteristics, quality, and results. A random-effects model was employed, and summary risk ratios (RR) with 95% confidence intervals (CI) were calculated.

**Results:**

A total of 468 patients from two randomized controlled trials and one cohort study were included in the systematic review and meta-analysis. Pooled results showed that septum resection did not improve the live birth rate for patients with a septate uterus (RR = 0.84, 95% CI = 0.56 – 1.25, P = 0.39). Additionally, no significant differences were found between the septum resection and expectant management groups in terms of clinical pregnancy (RR = 1.08, 95% CI 0.81 – 1.44, P = 0.60), abortion (RR = 1.99, 95% CI 0.80 – 4.98, P = 0.14), and preterm delivery rates (RR = 0.99, 95% CI 0.42 – 2.31, P = 0.98).

**Conclusion:**

Our data provide clear evidence that septum resection does not improve the reproductive outcomes of patients with a septate uterus. These findings might be useful for revising current clinical guidelines.

## Introduction

The uterine septum is the most common uterine anomaly, accounting for approximately 35% of detected Mullerian abnormalities (1). It is believed to develop from the incomplete resorption of the fused medial walls of the paramesonephric (Mullerian) ducts prior to the 20th embryonic week (2). Thus, a septate uterus exhibits a single fundus and an internal indentation (septum), which originates from the fundal midline and exceeds 50% of the uterine wall thickness, splitting the uterine cavity into two distinct parts (3).

The septate uterus has been associated with declining fertility (4). For example, the incidence of uterine septum is higher in women seeking treatment for subfertility than in the general population, implying an underlying association (5). Additionally, uterine septate has been regarded as a risk factor for miscarriage, as significant risk reduction following surgery has been demonstrated in studies where patients serve as their own internal controls (5). This evidence suggests that removal of the septum via surgery might be a potential approach to improving pregnancy outcomes.

Although the pathophysiology of the uterine septum in reproduction is unclear, it is reasonable to hypothesize that restoring normal anatomy might also improve its function. Initial approaches to incising the septum, such as Bret-Tompkins or Jones metroplasty, required a laparotomy (6, 7). Moreover, the advent of hysteroscopic septum resection, which offers a minimally invasive approach with a shorter recovery time, is now considered first-line therapy (8). Numerous retrospective studies have compared reproductive outcomes for patients with a septate uterus before and after the surgery, reporting superior outcomes in terms of pregnancy rates, preterm birth rates, and live births (9–12). However, this evidence has a high risk of bias due to the study design, with the same group of women serving as both the study and control groups, since before-and-after comparison research tends to favor the intervention (13). Additionally, research with positive results is more frequently published, contributing to publication bias. Thus, there is no solid evidence confirming the benefits of septum resection for patients with a septate uterus.

In the current study, a literature review and meta-analysis were conducted to obtain higher-grade evidence. Both cohort studies and randomized controlled trials were included to evaluate the reproductive outcomes of different treatments (septum resection or expectant management) for women with a septate uterus.

## Materials and methods

### Literature search

Studies were identified in the following electronic databases: Pubmed, Embase, and Cochrane Library, using the search terms: septal resection OR hysteroscopic metroplasty OR septum resection OR septate uterus OR uterine septum, with adjustments made for each database as necessary. The detailed search strategy is displayed in **Supplementary Table 1**. There were no restrictions on study design or language. The final research was conducted, including all publications appearing in the databases before 14 August. This systematic review was conducted according to the Preferred Reporting Items for Systematic Reviews and Meta-Analysis (PRISMA) Statement.

### Inclusion and exclusion criteria

The inclusion criteria were women with septate uteri and undergoing septum resection or expectant management. Moreover, the included studies had to report at least one of the following reproductive outcomes after treatment: clinical pregnancy, live birth, preterm delivery, term delivery, or abortion. Both randomized controlled trials and cohort studies (retrospective and prospective) published in English were included. Reviews, editorials, letters, case reports, case serials, animal experimental studies, conference abstracts, and articles in other languages were excluded.

### Study selection

Titles and abstracts of all identified publications were screened by two of the authors (C.L. and Z.L.). The full texts of the pre-selected articles were reviewed according to the inclusion and exclusion criteria. If consensus could not be reached, disagreements were settled through discussion with a third author (X.G.).

### Outcome measurement

All patients in the included studies were diagnosed with a septate uterus via 3D ultrasound, MRI, hysteroscopy, or hysterosalpingography. Following the diagnosis of a uterine septum, patients were expected to conceive naturally or with assisted reproductive technologies, either in the expectant management group or after septum resection. Women in both groups were followed up for 12 months if not pregnant. In addition, patients who conceived continued to be followed up until delivery or abortion. Clinical pregnancy was defined as the presence of a fetal heartbeat at or beyond 6 weeks of pregnancy. The spontaneous demise of a pregnancy, including non-visualized or biochemical pregnancies confirmed by serum or urine b-HCG, was considered abortion. Preterm delivery was defined as birth before a gestational age of complete weeks. The clinical pregnancy, live birth, abortion, and preterm delivery rates were calculated as the number of events that occurred divided by the number of included participants, respectively.

### Data extraction and risk of bias assessment

The following data were extracted from all eligible included studies by two of the authors (C.L. and X.G.): authors, year of publication, location of the study groups.

Study design, years of study, age, number of participants, length of follow-up, number of patients assigned to the two groups, and reproductive outcomes. Any disagreements were resolved by another investigator (Z.L.).

Two investigators (C.L. and Z.L.) independently evaluated the trials for risk of bias. The assessment was based on the criteria outlined in Chapter 8 of the Cochrane Handbook and included random sequence generation, allocation concealment, blinding of participants and personnel, blinding of outcome assessment, incomplete outcome data, selective reporting, and other biases (14). Each criterion was characterized as low, high, or unclear. Disagreement were resolved through discussion with another investigator (X.G.).

### Statistical analysis

All metadata analyses were conducted using Review Manager 5.3 (Cochrane Collaboration, Oxford, UK). Dichotomous variables were analyzed using a risk ratio (RR) with 95% confidence intervals (CIs) employing a random-effects model. Statistical heterogeneity was quantified using the Chi-squared and I^2^ statistics. A value of I^2^ greater than 50%, or P < 0.05, signified significant heterogeneity (15). To assess publication bias, a funnel plot analysis using the Egger test was performed. The results were presented as forest plots. A significance level of P < 0.05 was considered statistically significant.

## Results

### Study selection and characteristics

**Figure 1:**
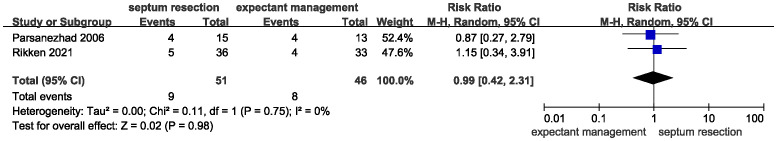
Flow diagram of the study identification and selection process for systematic review and meta-analysis.

**Figure 2:**
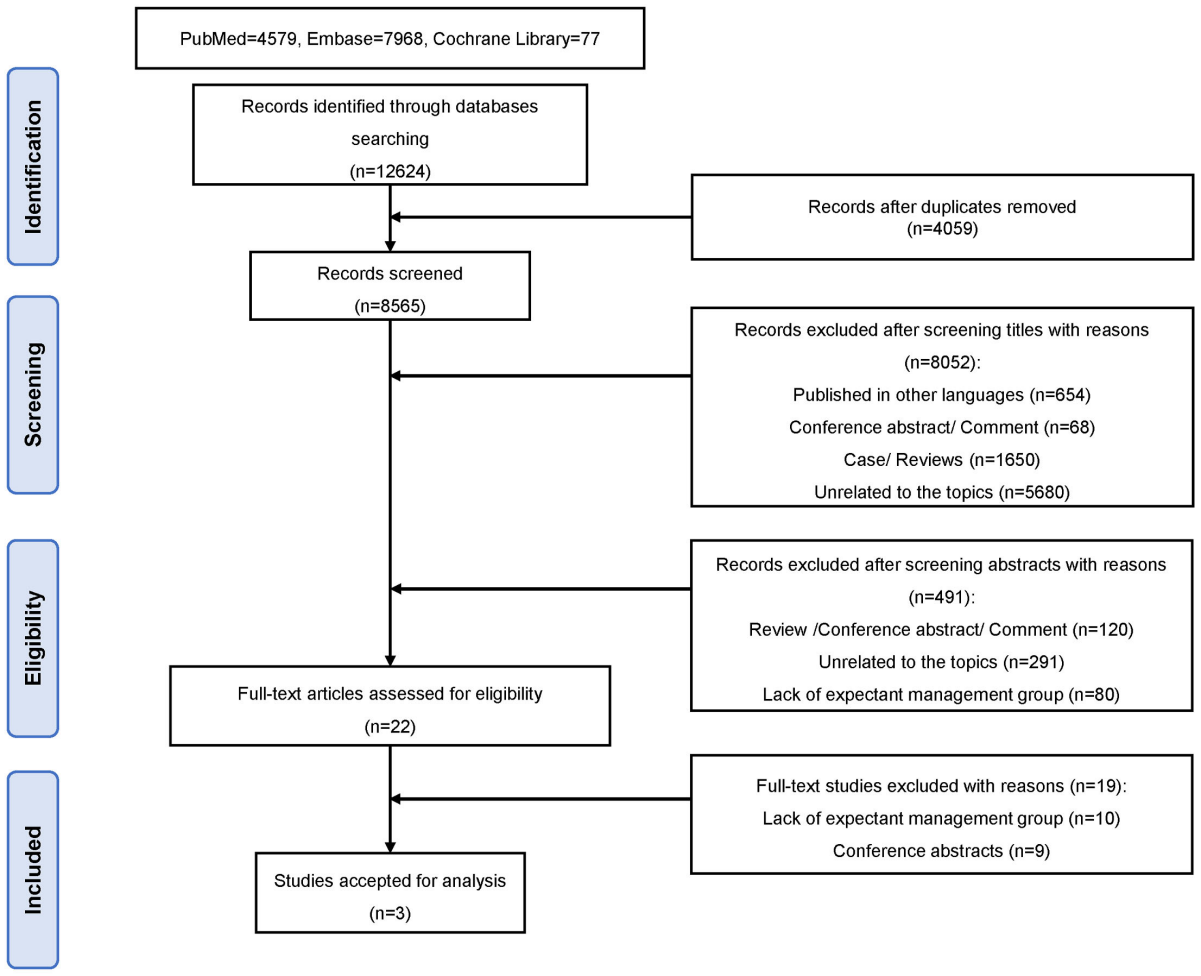
Risk of bias summary table.

The detailed selection process for studies is documented in a PRISMA (Preferred Reporting Items for Systematic Reviews and Meta-Analyses) flow diagram (**Figure 1**). The literature search yielded a total of 8,565 publications after the removal of duplicates. After reviewing the titles and abstracts, 22 records were assessed for eligibility by full-text screening. Of these, 10 studies were ineligible because they lacked an expectant management group, and 9 were conference abstracts. Finally, three studies met the inclusion criteria and were included in the current meta-analysis (16–18). The risk of bias summary for the included trials is displayed in **Figure 2**.

The characteristics of the eligible studies are displayed in **Table 1**. Two of them were randomized controlled trials, and the other was a retrospective cohort study. The investigation periods ranged from 6 years to 18 years, and the length of follow-up ranged from 12 months to 53 months. A total of 468 patients from three studies were enrolled in this meta-analysis. All three studies recruited patients from multiple centers.

**Table 1:** Characteristics of the included studies.

Author, Year	Location	Study design	Years of study	Age	Patients(N)	Length of follow-up	Septum resection (N)	Expectant management (N)	Reproductive outcomes
Parsanezhad, 2006 (16)	Iran and Germany	randomized controlled trial	1999–2005	18–35	132	12 months	15	13	Pregnancy, abortion, preterm delivery, live birth, and cesarean rates
Rikken, 2020 (17)	Netherlands, USA, and UK	Retrospective cohort	2000–2018	/*	257	Up to 53 months	151	106	Conception, live birth, ongoing pregnancy, abortion, and preterm birth rates
Rikken, 2021 (18)	Netherlands, UK, USA, and Iran	randomized controlled trial	2010–2018	29–33	79	12 months	36	33	Live birth, ongoing pregnancy, clinical pregnancy, abortion, and preterm birth rates

*the age of the included participants was displayed as the mean (SD) in the original study. To be specific, the average age of the participants was 31.7 years (4.18) in the septum resection group and 30.8 years (5.09) in the expectant management group, respectively.

### Primary outcome

**Figure 3:**
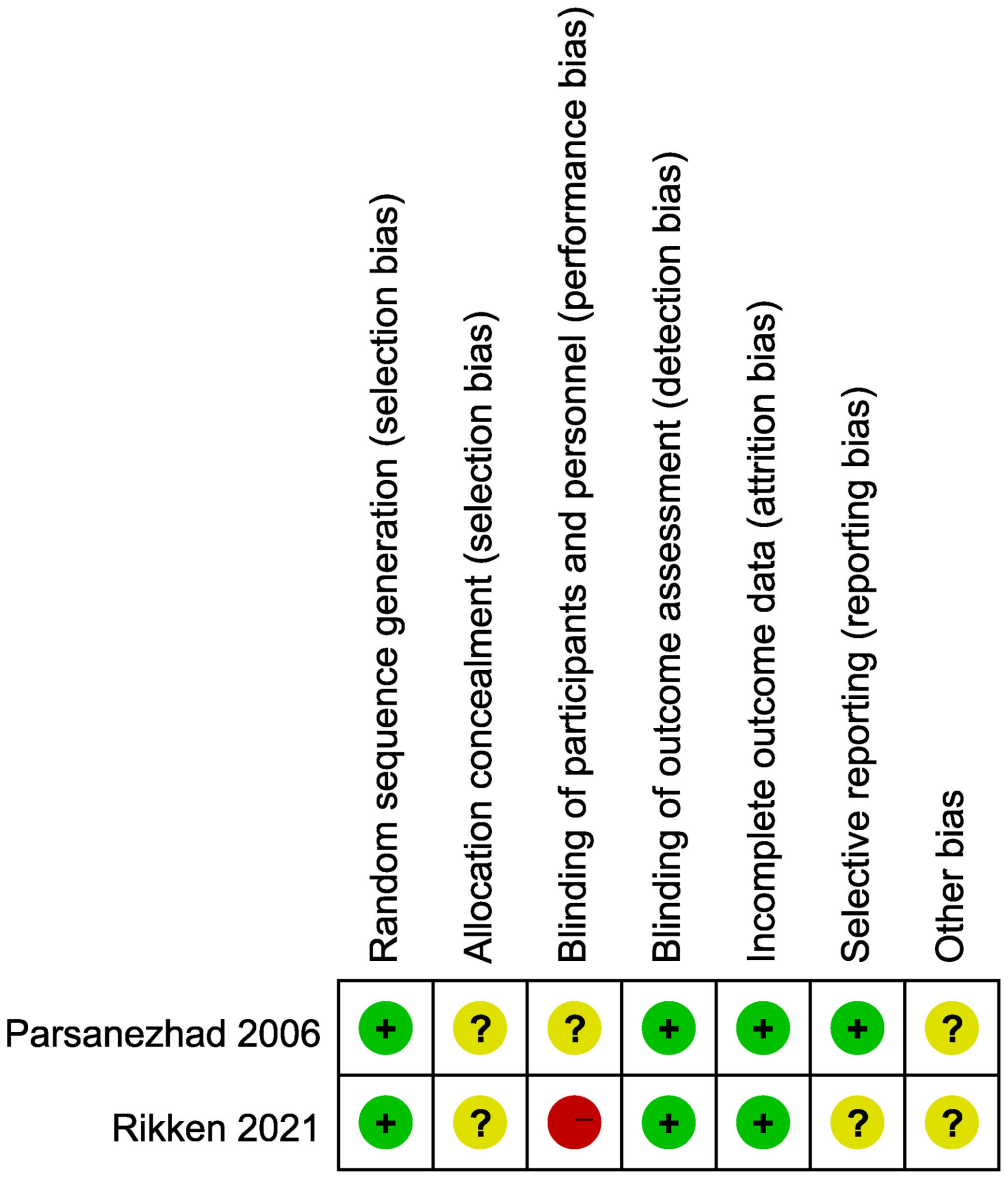
Forest plots of the live birth rate in patients with a septate uterus receiving septum resection and expectant management.

All studies provided data for the primary outcome of live birth. There was heterogeneity for this outcome among studies, as indicated by the I^2^ value (I^2^ = 61%). The pooled results indicated that incising the uterine septum could improve the live birth rate compared with expectant management (**Figure 3**, RR = 0.84, 95% CI 0.56 – 1.25, P = 0.39). Based on these data, septum resection was not conclusively suggested for women with a septate uterus and a desire to conceive.

### Secondary outcomes

**Figure 4:**
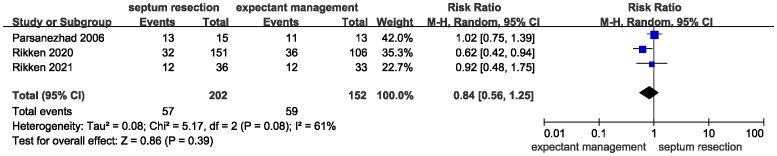
Forest plots of the clinical pregnancy rate in patients with a septate uterus receiving septum resection and expectant management.

**Figure 5:**
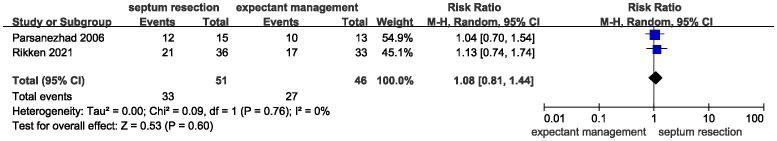
Forest plots of the abortion rate in patients with a septate uterus receiving septum resection and expectant management.

**Figure 6:**
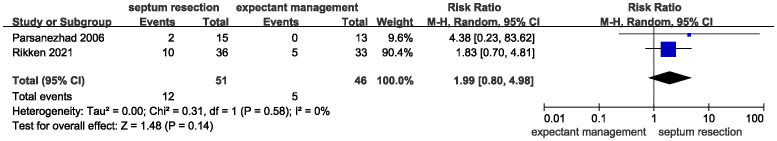
Forest plots of the preterm delivery rate in patients with a septate uterus receiving septum resection and expectant management.

Results for secondary outcomes, including clinical pregnancy, abortion, and preterm delivery, showed no significant heterogeneity. Additionally, there were no significant differences between the two groups regarding the clinical pregnancy rate (**Figure 4**, RR = 1.08, 95% CI 0.81 – 1.44, P = 0.60, heterogeneity: I^2^ = 0%, P = 0.76), abortion rate (**Figure 5**, RR = 1.99, 95% CI 0.80 – 4.98, P = 0.14; heterogeneity: I^2^ = 0%, P = 0.58), and preterm delivery rate (**Figure 6**, RR = 0.99, 95% CI 0.42 – 2.31, P = 0.98; heterogeneity: I^2^ = 0%, P = 0.75).

### Publication bias

Funnel plots for publication bias included in different treatment groups are shown in **Supplementary Figures 1**-**4**. The results showed no evidence of significant publication bias, as the Egger test was not significant.

## Discussion

In this study, we identified two trials and one retrospective study that compared reproductive outcomes between septum resection and expectant management for women with a septate uterus. A total of 468 patients from three studies were included in the meta-analysis. Summary RRs indicated that incising the septum did not increase the chance of live birth and clinical pregnancy rates, nor did it decrease the risk of adverse obstetric outcomes, such as abortion rate and preterm birth rate. Based on these results, patients with a septate uterus may not gain any improvements in reproductive outcomes from septum resection, questioning the rationale behind the surgery.

Currently, incising the septum via hysteroscopy has been recognized as an effective approach to improving reproduction, as suggested by multiple studies (5, 19). However, the results of this meta-analysis indicated that no differences were found between septum resection and expectant management, which was in line with a previous retrospective cohort study (17). However, such findings seem to contradict the results of prior observational research with a before/after study design, which reported significant improvements in live birth and clinical pregnancy rates after surgery (20). We speculate on two possibilities for this divergence. First, the study design of such observational research was “before/after,” which always favors the tested intervention. Second, the conclusions of these studies could be limited by their retrospective nature. These non-randomized comparative studies did not accurately account for confounders, and some were also at high risk of selection bias. For example, there was an unequal distribution of patients in a previous study, with 109 in the surgery group and 15 in the control group (21).

In fact, incising the uterine septum without improvements in fecundity is not surprising. When women experiencing infertility present to a reproductive center without identifiable risks, such as a uterine septum, there may be pressure from both the provider and patient to pursue immediate resection based on the stereotype that restoring normal anatomy also restores normal function (1, 3). The conventional view holds that the main composition of the uterine septum was fibromuscular tissue, with more connective tissues and fewer muscular fibers (22, 23). However, this assumption contradicts histological findings that the muscle bundles accounted for over 50% of the septum (24). Besides, the linear arrangement of smooth muscle and vessels in the core of the septa is similar to that of the normal myometrium (25). Thus, metroplasty corrects uterine anatomy while also injuring the inner face of the myometrium and the endometrium, which may take considerable time for recovery (26). In this study, the follow-up period from surgery to pregnancy was only 12 months, which might be too short for functional recovery of the uterus. Therefore, there were no significant differences in clinical pregnancy and live birth rates between the two treatments, which is reasonable.

To date, studies comparing the prognosis of uterine septum resection or expectant management are limited. This review, to our best knowledge, is the first meta-analysis to address these issues. Moreover, this study provided high-quality evidence and raised questions about routine hysteroscopic septum resection for women with a septate uterus. However, this meta-analysis was limited by the lack of trials on the topic. After literature selection, only two trials were included in the meta-analysis, reducing its potential impact. Besides, owing to the small sample size, the included studies could not evaluate the differential effects of septum resection in women with pregnancies compared to those presenting with subfertility. Therefore, large-scale studies with subgroup analysis for patients with different conditions are required to confirm the effectiveness of uterine septum resection on reproduction.

## Data availability statement

The raw data supporting the conclusions of this article will be made available by the authors, without undue reservation.

## Author contributions

CL: Conceptualization, Writing – original draft. ZL: Investigation, Writing – original draft. XG: Investigation, Writing – original draft. YC: Writing – review & editing.

## Supplementary material

Supplementary Figure 1 Funnel plots for publication bias evaluation for the live birth rate for women who underwent septum resection versus expectant management.

Supplementary Figure 2 Funnel plots for publication bias evaluation for the clinical pregnancy rate for women who underwent septum resection versus expectant management.

Supplementary Figure 3 Funnel plots for publication bias evaluation for the abortion rate for women who underwent septum resection versus expectant management.

Supplementary Figure 4 Funnel plots for publication bias evaluation for the preterm birth rate for women who underwent septum resection versus expectant management.

Supplementary Table 1 The detailed search algorithm used in electronic databases.
