# Supplementary figures and images for: Does septum resection improve reproductive outcomes for women with a septate uterus? A systematic review and meta-analysis
Source: Front Endocrinol (Lausanne). 2024 Jul 22;15:1361358. doi: 10.3389/fendo.2024.1361358 (PMC11298444; doi:10.3389/fendo.2024.1361358)

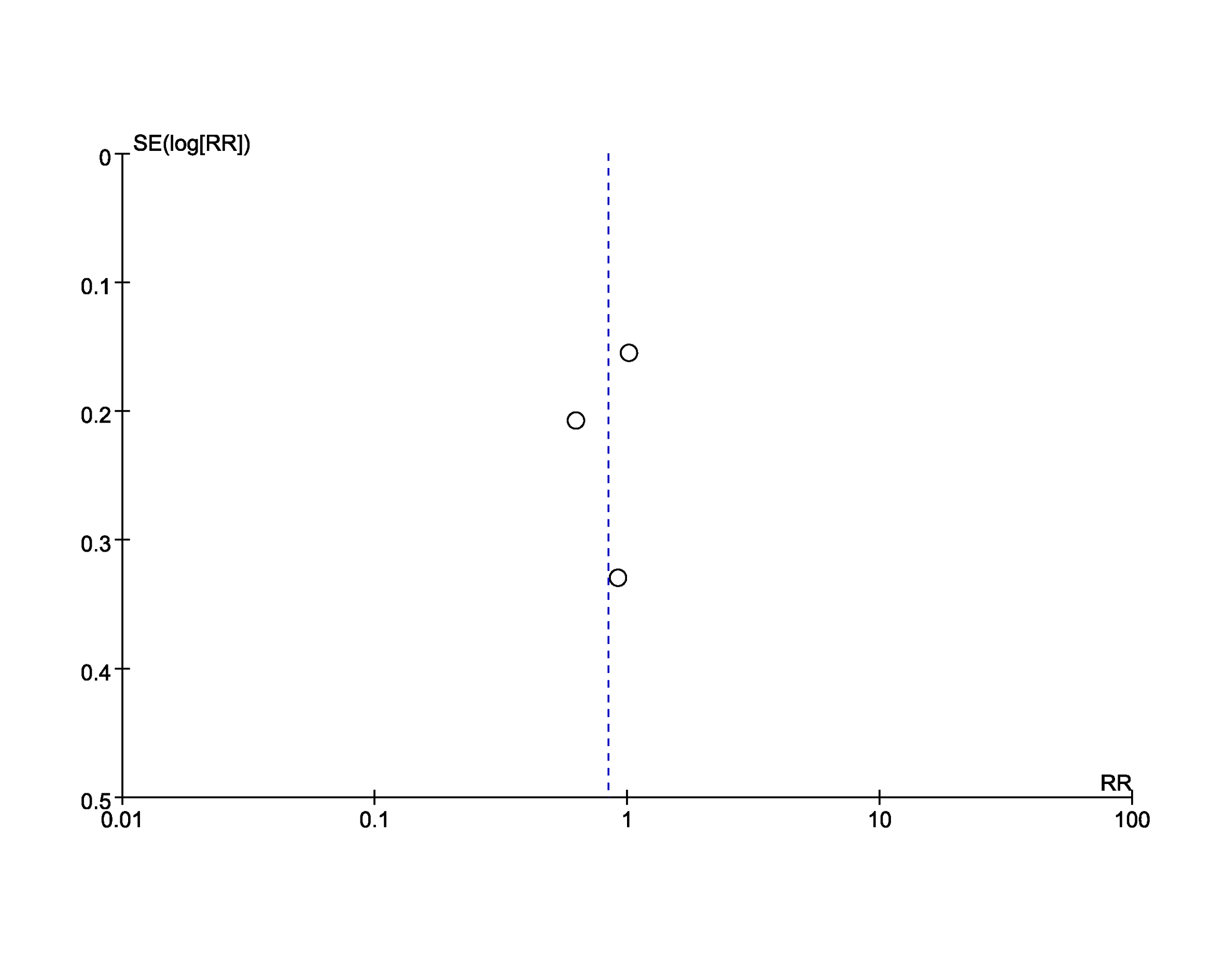

Supplement: Supplementary Figure 1 — Funnel plots for publication bias evaluation for the live birth rate for women who underwent septum resection versus expectant management. [file Image_1.tif]

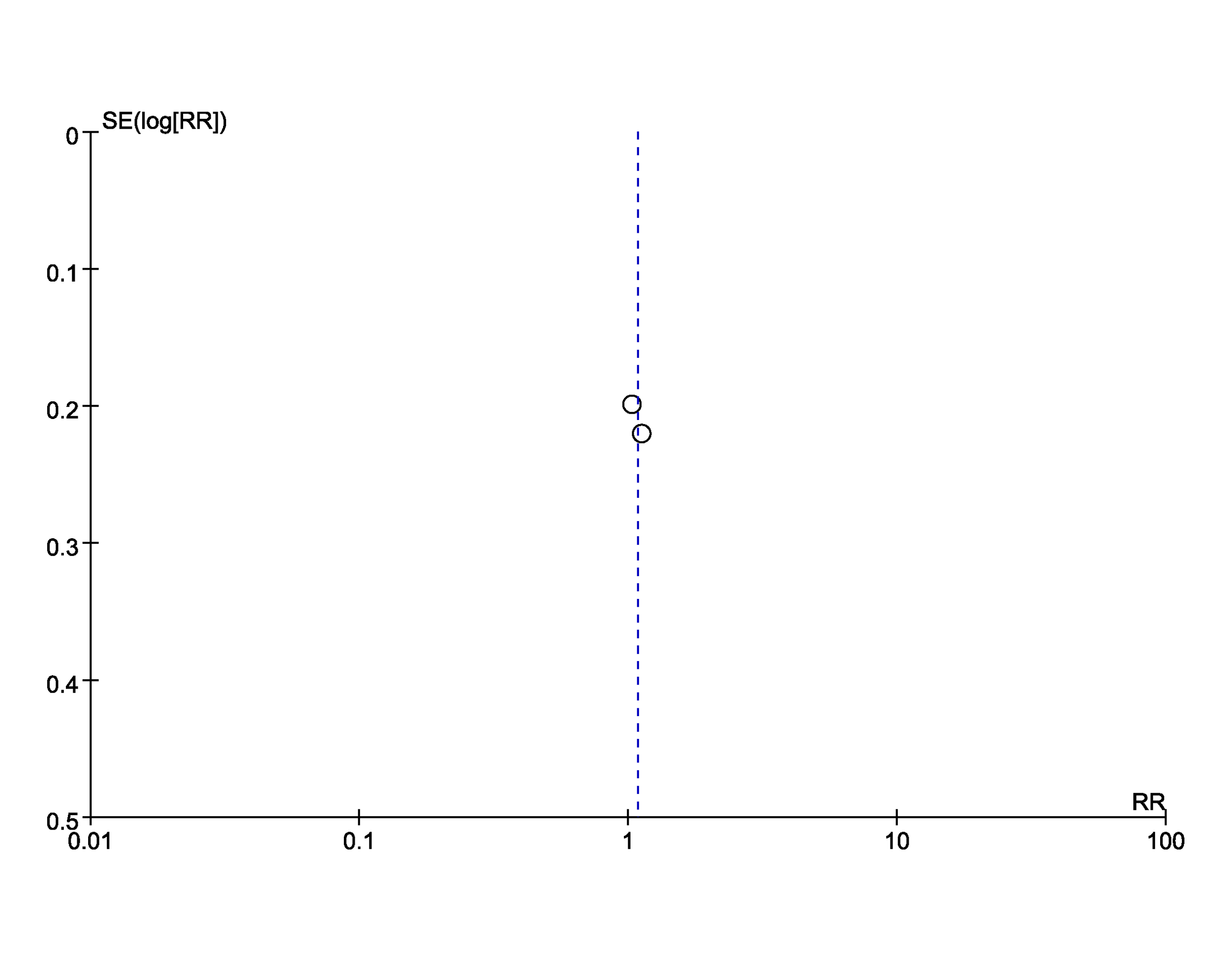

Supplement: Supplementary Figure 2 — Funnel plots for publication bias evaluation for the clinical pregnancy rate for women who underwent septum resection versus expectant management. [file Image_2.tif]

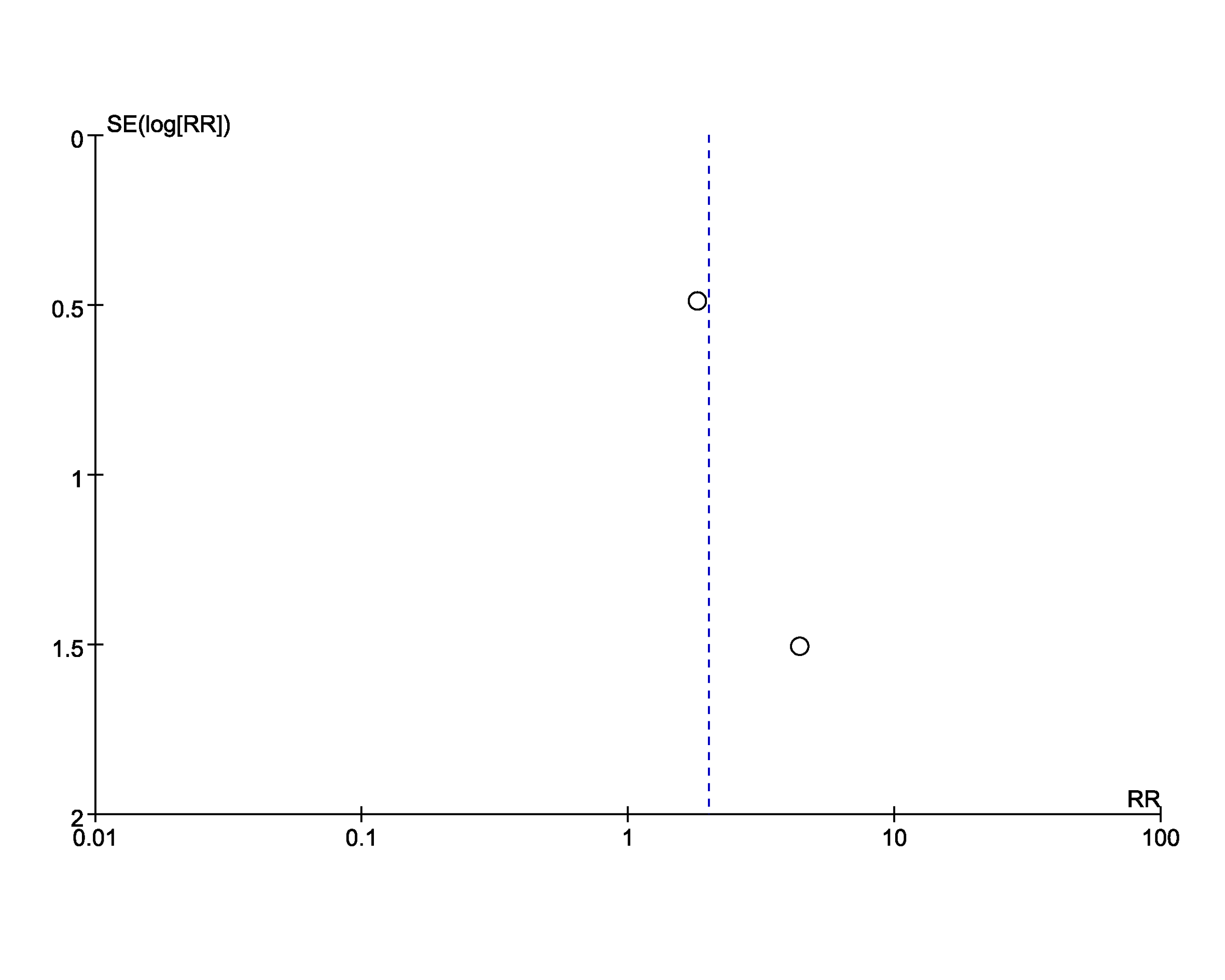

Supplement: Supplementary Figure 3 — Funnel plots for publication bias evaluation for the abortion rate for women who underwent septum resection versus expectant management. [file Image_3.tif]

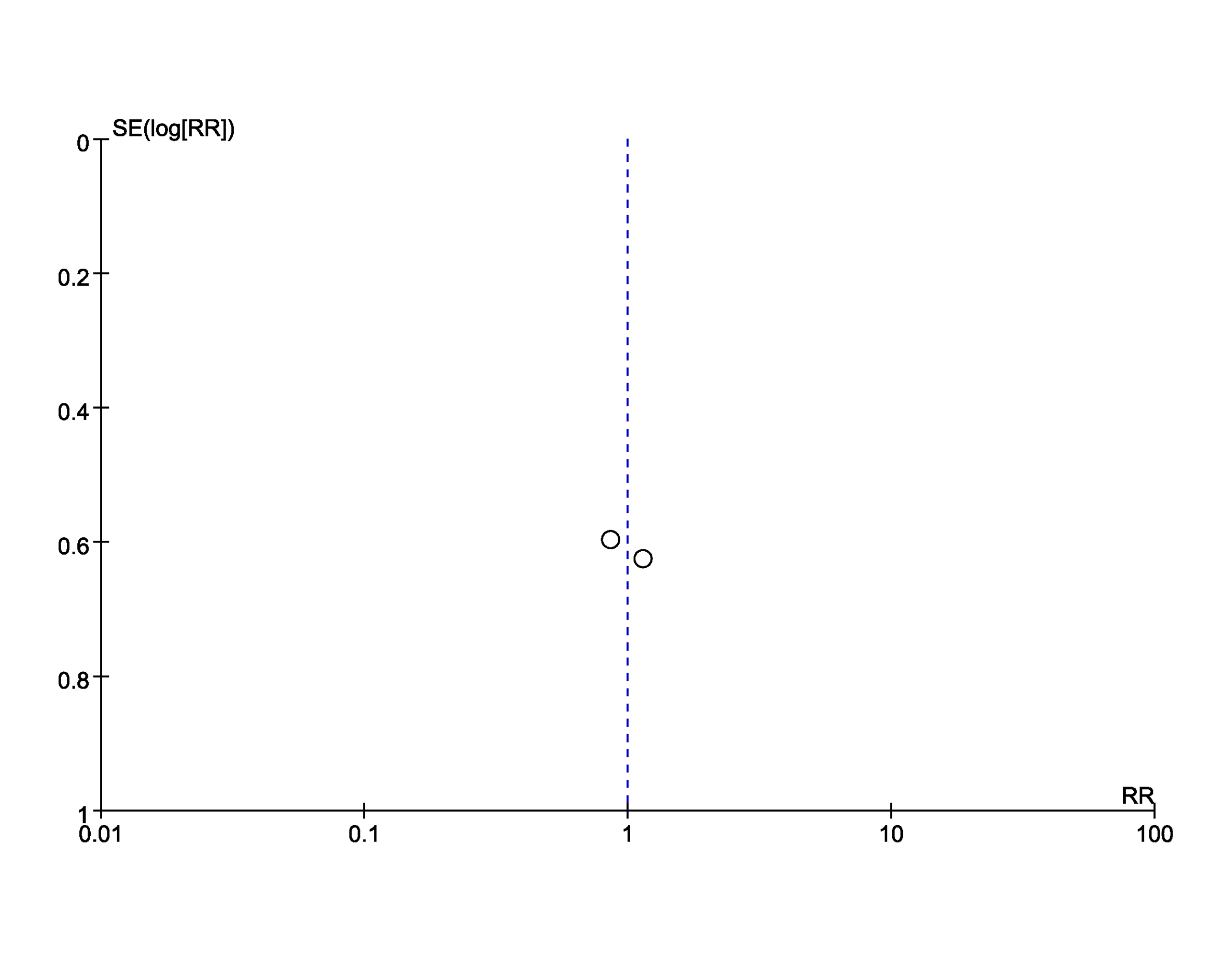

Supplement: Supplementary Figure 4 — Funnel plots for publication bias evaluation for the preterm birth rate for women who underwent septum resection versus expectant management. [file Image_4.tif]
