# Supplementary material for: Does septum resection improve reproductive outcomes for women with a septate uterus? A systematic review and meta-analysis
Source: Front Endocrinol (Lausanne). 2024 Jul 22;15:1361358. doi: 10.3389/fendo.2024.1361358 (PMC11298444; doi:10.3389/fendo.2024.1361358)
Supplement: Supplementary Table 1 — The detailed search algorithm used in electronic databases. [file Table_1.docx]

**Supplemental Table 1 The detailed searching algorithm used in electronic databases**

| Dataset | Search strategy |
| --- | --- |
| Pubmed | ((((septal resection) OR (hysteroscopic metroplasty)) OR (septum resection)) OR (septate uterus)) OR (uterine septum) |
| Embase | (uterine AND septum) OR (septate AND uterus) OR (septum AND resection) OR (hysteroscopic AND metroplasty) OR (septal AND resection) |
| Cochrane library | (uterine septum):ti,ab,kw OR (septate uterus):ti,ab,kw OR (septum resection):ti,ab,kw AND (hysteroscopic metroplasty):ti,ab,kw AND (septal resection):ti,ab,kw |
